# Nrf2 Ablation Promotes Alzheimer's Disease-Like Pathology in APP/PS1 Transgenic Mice: The Role of Neuroinflammation and Oxidative Stress

**DOI:** 10.1155/2020/3050971

**Published:** 2020-05-11

**Authors:** Peng Ren, Jingwei Chen, Bingxuan Li, Mengzhou Zhang, Bei Yang, Xiangshen Guo, Ziyuan Chen, Hao Cheng, Pengfei Wang, Shuaibo Wang, Ning Wang, Guohua Zhang, Xu Wu, Dan Ma, Dawei Guan, Rui Zhao

**Affiliations:** ^1^Department of Forensic Pathology, School of Forensic Medicine, China Medical University, Shenyang, Liaoning 110122, China; ^2^Department of Forensic Medicine, Criminal Investigation Police University of China, Shenyang, Liaoning 110854, China; ^3^Remote Forensic Consultation Center, Collaborative Innovation Center of Judicial Civilization, China University of Political Science and Law, Beijing 100192, China; ^4^Department of Histology and Embryology, School of Basic Medicine, China Medical University, Shenyang, Liaoning 110122, China; ^5^Dalian Municipal Women and Children's Medical Center, Dalian, Liaoning 116037, China

## Abstract

**Introduction:**

Alzheimer's disease (AD), the most common neurodegenerative disorder, is characterized by the accumulation of amyloid-*β* (A*β*) peptide and hyperphosphorylated tau protein. Accumulating evidence has revealed that the slow progressive deterioration of AD is associated with oxidative stress and chronic inflammation in the brain. Nuclear factor erythroid 2- (NF-E2-) related factor 2 (Nrf2), which acts through the Nrf2/ARE pathway, is a key regulator of the antioxidant and anti-inflammatory response. Although recent data show a link between Nrf2 and AD-related cognitive decline, the mechanism is still unknown. Thus, we explored how Nrf2 protects brain cells against the oxidative stress and inflammation of AD in a mouse model of AD (APP/PS1 transgenic (AT) mice) with genetic removal of Nrf2.

**Methods:**

The spatial learning and memory abilities of 12-month-old transgenic mice were evaluated using a Morris water maze test. Hippocampal levels of Nrf2, A*β*, and p-tauS404 and of astrocytes and microglia were determined by immunostaining. Inflammatory cytokines were determined by ELISA and quantitative real-time polymerase chain reaction (qRT-PCR). Oxidative stress was measured by 8-hydroxydeoxyguanosine immunohistochemistry, and the antioxidant response was determined by qRT-PCR.

**Results:**

The spatial learning and memory abilities of AT mice were impaired after Nrf2 deletion. A*β* and p-tauS404 accumulation was increased in the hippocampus of AT/Nrf2-KO mice. Astroglial and microglial activation was exacerbated, followed by upregulation of the proinflammatory cytokines IL-1*β*, IL-6, and TNF-*α*.

**Conclusion:**

Our present results show that Nrf2 deficiency aggravates AD-like pathology in AT mice. This phenotype was associated with increased levels of oxidative and proinflammatory markers, which suggests that the Nrf2 pathway may be a promising therapeutic target for AD.

## 1. Introduction

Alzheimer's disease (AD) is a progressive cognitive dysfunction that is pathologically characterized by aggregated amyloid-*β* (A*β*) plaques and neurofibrillary tangles (NFTs) consisting of hyperphosphorylated tau [1, 2]. A*β* aggregation can be triggered by mutation and overexpression of A*β* precursor protein (APP), presenilin 1, or presenilin 2 genes [3]. Accumulating evidence has shown that oxidative, inflammatory, and metabolic stress might precede the proteinopathy in the prodromal and early stages of the disease [4, 5]. The neurotoxicity of A*β* deposition in the brain causes oxidative stress and neuronal damage, which are followed by an inflammatory response [6]. Meanwhile, oxidative stress and inflammation play important roles in the neurodegenerative process of AD [7].

Nuclear factor erythroid 2- (NF-E2-) related factor 2 (Nrf2) is a transcription factor that regulates the transcription of numerous phase II detoxifying and antioxidant enzymes, such as hemeoxygenase-1 (HO-1) and NAD (P)H: quinine oxidoreductase 1 (NQO1), via the antioxidant response element (ARE) pathway [8], as well as that of proinflammatory cytokines, by opposing the transcriptional upregulation of these genes [9]. Nrf2 activation protects our bodies from detrimental stress by upregulating the antioxidative defense pathway, inhibiting inflammation, and maintaining protein homeostasis. The Nrf2 level is reported to decrease as a function of age [10] and to be lower in the postmortem human brains and animal models of AD [11, 12]. Recent studies also indicated a correlation between Nrf2 deficits and AD [10, 13, 14], not only because the transcription factor helps to mitigate oxidative stress and inflammation but also because it directly and indirectly regulates changes in autophagy in vivo and in vitro [15–18]. Some studies have noted that Nrf2 ablation exacerbates cognitive deficits in a mouse model of AD and aggravates AD-like pathology [13, 14, 18]. Nrf2 activation through genetic and pharmaceutical approaches exerts a neuroprotective role in AD-related pathophysiology [19, 20], but the effect of Nrf2 silencing on the pathogenesis of AD-like dysfunction remains elusive.

In the present study, we used a genetic approach to delete the Nrf2 gene from APP/PS1 transgenic (AT) mice, a widely used animal model of AD displaying impaired cognitive function at 9 months old [21]. We found that the ablation of Nrf2 in AT mice exacerbated spatial learning and memory deficits and AD-like pathology. These changes were correlated with oxidative damage and neuroinflammatory processes.

## 2. Materials and Methods

### 2.1. Animals

AT mice (on a C57BL/6J background, Jackson Laboratory, stock number 034832) and Nrf2 gene knockout (Nrf2-KO) mice (on a C57BL/6 background, originally generated by Dr. Masayuki Yamamoto) were used in this work. In line with the strategy described in the literature [13], we generated four genotypes: wild-type (WT) (APP/PS1^−/−^, Nrf2^+/−^, *n* = 4 males and 2 females), Nrf2*-*KO (APP/PS1^−/−^, Nrf2^−/−^, *n* = 6 males and 2 females), AT (APP/PS1^+/−^, Nrf2^+/−^, *n* = 6 males and 6 females), and AT/Nrf2*-*KO **(**APP/PS1^+/−^, Nrf2^−/−^, *n* = 6 males and 7 females). The animals used and analyzed in this study were littermates and 12 months old, and both male and female mice were used. Animals were housed 3-5 per cage at room temperature on a 12 h light : dark cycle. Rodent diet and water were provided ad libitum under SPF (specific pathogen free) conditions. All mice were genotyped through polymerase chain reaction (PCR) amplification of genomic DNA extracted from tail snips (Figure S1(A)). The Nrf2 mRNA level in the mouse brain was also assessed by quantitative real-time PCR (qRT-PCR) after sample collection (Figure S1(D)). All experiments were conducted in accordance with protocols approved by the China Medical University Animal Care and Use Committee.

### 2.2. Morris Water Maze Test

A Morris water maze test was used to assess mouse spatial learning and memory abilities. It was performed as previously described [22]. Briefly, the experiments were conducted in three stages: a cued learning test, a navigation test, and a probe test. In the cued learning test stage (1st day), the escape platform was placed above the surface of the water. In the navigation test stage (2nd, 3rd, 4th, and 5th day), the escape platform was placed below the surface of the water. The mice underwent four training trials per day for 4 consecutive days to allow them to find the hidden platform; the escape latency and distance traveled were scored as an indication of learning. In the probe test stage (6th day), the escape platform was removed and the mice were supposed to swim in the pool for 60 s. Two consecutive tests were implemented at 15 min intervals. Several dependent variables were scored—the swim distance to the platform quadrant, the latency to the platform quadrant, the frequency of crosses to the platform quadrant, and the frequency of crosses to the platform—and analyzed by the SMART™ tracking software (San Diego Instruments, San Diego, CA, USA).

### 2.3. Samples Collection

The mice were anesthetized with intraperitoneal injection of pentobarbital sodium and perfused with cold PBS (phosphate buffer saline) for 5 min. After perfusion, the mice brains were removed and bisected sagittally. Half of the brain was fixed in 4% paraformaldehyde, the other half was dissected to obtain hippocampus and stored at −80°C until use. We used the fixed tissue for the staining experiments of immunohistochemistry and immunofluorescence. The frozen hippocampus was used for protein and RNA extraction. The information of total mice and their use for various protocols are listed in Table S1.

### 2.4. Immunohistochemistry and Immunofluorescence

Immunohistochemical staining was performed on formalin-fixed and paraffin-embedded mouse brain tissue sections as previously described [23]. Briefly, 4 *μ*m thick sections were deparaffinized with alcohol and washed twice with PBS (pH = 7.4) and then treated with 0.3% H_2_O_2_ in methanol to block the endogenous peroxidase activity. After microwave antigen retrieval with 0.01% mmol/L citrate buffer (pH 6.0) for 5 min, the sections were washed with PBS. Then 5% normal serum was used to block the nonspecific binding for 1 h followed by incubation with primary antibody (Table 1) diluted in PBS overnight at 4°C. To remove the primary antibody, the sections were washed and then incubated with suitable biotinylated secondary antibody for 30 min at room temperature. After being washed with PBS, the sections were incubated with streptavidin peroxidase and visualized with diaminobenzidine (DAB). Then the sections were routinely counterstained with hematoxylin. Images were obtained with an Olympus microscope (BX51) at 400× magnification.

For immunofluorescence staining, after microwave antigen retrieval, the sections were blocked with 5% bovine serum albumin (BSA) and incubated with diluted primary antibody (Table 1) overnight at 4°C. To remove the primary antibody, the sections were washed and then incubated with the appropriate Alexa Fluor®-labeled secondary antibody (Table 1) at room temperature for 2 h. After phosphate-buffered saline (PBS) washes, the nuclei were stained with DAPI (Sigma Chemical Company). Images were obtained with a Leica microscope (DMi8) at 400× magnification.

We localized the hippocampus in the mice brain by coronal brain matrices. Coronal section with the same-shape hippocampus from six different mice were selected, and five fields images were randomly taken without overlap in the hippocampus (i.e., CA1, CA3, and dentate gyrus). For the quantification analysis, the investigators were blind to genotype, and images selection and data acquisition were made randomly. Images were analyzed with ImageJ.

### 2.5. Enzyme-Linked Immunosorbent Assay

Cytokine content, including interleukin- (IL-) 1*β*, IL-6, IL-4, IL-10, tumor necrosis factor- (TNF-) *α*, and transforming growth factor- (TGF-) *β*, was determined using commercially available enzyme-linked immunosorbent assay (ELISA) kits (MM-0040M2, MM-0163M2, MM-0165M2, MM-0176M1, MM-0689M2, and MM-0132M2; MeiMian, Wuhan, China). Assays were performed according to the manufacturer's instructions. Briefly, the hippocampal tissue was homogenized in PBS and centrifuged at 12,000 × *g* three times for 30 min at 4°C to remove precipitate. The samples were incubated for 2 h at 37°C in the antibody-precoated ELISA plates. The wells were washed 3 times, followed by incubation for 1.5 h at 37°C with peroxidase-conjugated antibody solution. They were colored with TMB for 20 min at 37°C, and the reaction was stopped with 2 mol/L H_2_SO_4_. The OD value was measured with ELx808 at 450 nm. The results were averaged and expressed as nanograms per liter.

### 2.6. Quantitative Real-Time PCR

Total RNA was extracted from frozen hippocampal tissue using TRIzol reagent (10296-028; Thermo Fisher Scientific) according to the manufacturer's instructions. The OD_260_ and the OD_260_/OD_280_ ratio were assessed to determine the concentration and purity, respectively, of each RNA sample via ultraviolet spectrophotometry. RNA was reversely transcribed into cDNA using the PrimeScript™ RT reagent kit (RR037A; Takara Biotechnology). The cDNA was synthesized in a 10 *μ*L reaction volume and used in qRT-PCR amplification with sequence-specific primer pairs. The primer pairs included Nrf2, IL-1*β*, IL-4, IL-6, IL-10, TNF-*α*, TGF-*β*, HO-1, NQO1, GCLC, GCLM, and GAPDH (Table 2). The qRT-PCR was performed with LightCycler® 480 II (Roche) using the SYBR® Premix Ex Taq™ II RT-PCR Kit (RR820A; Takara Biotechnology). The thermal cycling parameters were as follows: 1 cycle at 95°C for 30 s, followed by 40 cycles of 95°C for 5 s and 60°C for 34 s for fluorescence signal acquisition. The internal control was GAPDH. The Ct value was inversely proportional to the original template number. Relative quantification was calculated using the comparative Ct (2^−*ΔΔ*Ct^) method, in which the ΔCt value reflects the difference in Ct between the target genes and the internal reference gene.

### 2.7. Statistical Analysis

All results were analyzed by GraphPad Prism and are presented as the mean ± standard deviation. *P* values were calculated by a *t*-test and one-way ANOVA. *P* < 0.05 was considered to be statistically significant.

## 3. Results

### 3.1. Nrf2 Expression Is Higher in the Hippocampus in AT Mice

Immunohistochemical staining of Nrf2 was performed in AT mice and their WT littermates. The Nrf2 staining density was higher in the CA1, CA3, and dentate gyrus hippocampal areas of AT mice than those of WT mice (Figure S1(B, C)), but the positive immunostaining largely localized to the cytoplasm in the hippocampal neurons and was weak in the nucleus. In addition, the Nrf2 mRNA level was higher in AT mice than in WT mice (Figure S1(C)).

### 3.2. Nrf2 Deficiency Aggravates Spatial Learning and Memory Damage in AT Mice

**Figure 1:**
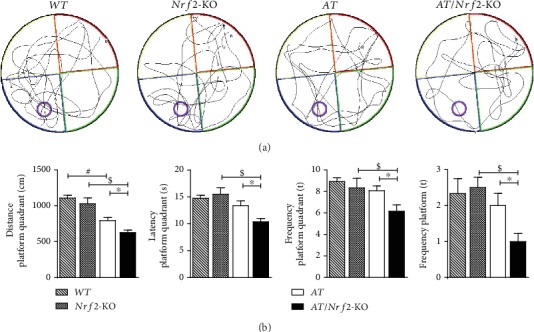
Nrf2 deficiency impaired spatial learning and memory in APP/PS1 mice illustrated by the Morris water maze. (a) Tracks of representative mice from the probe trial were shown among group (WT (APP/PS1^−/−^, Nrf2^+/−^, *n* = 4 males and 2 females), Nrf2-KO (APP/PS1^−/−^, Nrf2^−/−^, *n* = 4 males and 2 females), AT (APP/PS1^+/−^, Nrf2^+/−^, *n* = 3 males and 3 females), and AT/Nrf2-KO (APP/PS1^+/−^, Nrf2^−/−^, *n* = 3 males and 3 females). (b) There was a significantly difference in the swim distance to the platform quadrant, the latency to the platform quadrant, the frequency of crosses to the platform quadrant, and the frequency of crosses to the platform between AT mice, AT/Nrf2-KO mice, Nrf2-KO mice, and AT/Nrf2-KO mice (*n* = 6/genotype, ^$^^∗^*P* < 0.05). The swim distance to the platform quadrant was also significantly different between WT mice and AT mice (*n* = 6/genotype, ^#^*P* < 0.05). Data is represented as means ± SD and was analyzed by one-way ANOVA (b).

To evaluate the effects of Nrf2 on cognitive function in AT mice, we analyzed spatial learning and memory abilities in 12-month-old AT (APP/PS1^+/−^, Nrf2^+/−^) and AT/Nrf2-KO (APP/PS1^+/−^, Nrf2^−/−^) mice using a Morris water maze.  Figure 1(a) shows the representative probe tracks of mice. We found that the swim distance to the platform quadrant, the latency to the platform quadrant, the frequency of crosses to the platform quadrant, and the frequency of crosses to the platform were significantly different (*P* < 0.05) between AT mice and AT/Nrf2-KO mice (Figure 1(b)). Similarly, the swim distance to the platform quadrant, the latency to the platform quadrant, the frequency of crosses to the platform quadrant, and the frequency of crosses to the platform were also significantly different (*P* < 0.05) between Nrf2-KO mice and AT/Nrf2-KO mice (Figure 1(b)). The swim distance to the platform quadrant was also significantly different (*P* < 0.05) between WT mice and AT mice (Figure 1(b), left panel). Our data suggest that Nrf2 deletion aggravates the damage to spatial learning and memory abilities in AT mice.

### 3.3. Immunohistochemical Staining of A*β* and p-tauS404 in the Hippocampus Is Enhanced with Nrf2 Ablation in AT Mice

**Figure 2:**
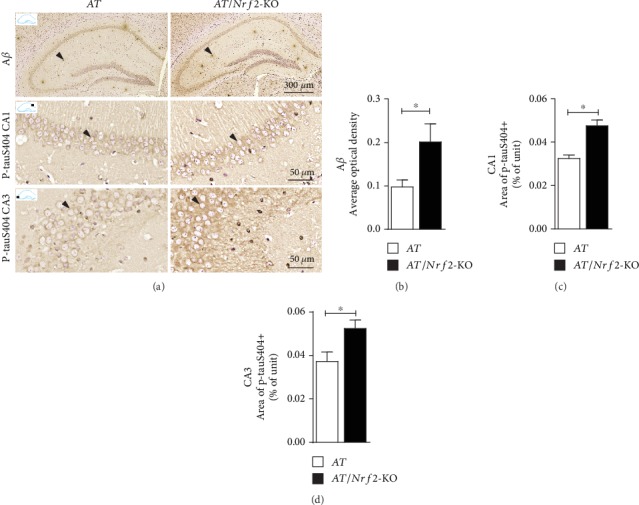
Nrf2 deficiency increased amyloid-beta (A*β*) deposits and phosphorylated tau protein (p-tauS404) expression in the hippocampus of APP/PS1 mice. (a) Immunohistochemical staining with anti-A*β* (scale = 300 *μ*m) and anti-p-tauS404 (scale = 50 *μ*m) in the hippocampus (arrow showed neuron). (b) A significant increase of average optical density of A*β* immunoreactivity in AT/Nrf2-KO genotype mice hippocampus (*n* = 5/genotype, ^∗^*P* < 0.05). (c, d) We found a significant difference for the area of p-tauS404 positive cells in CA1 and CA3 (*n* = 5/genotype, ^∗^*P* < 0.05). Data is represented as means ± SD and was analyzed by *t*-test (b–d).

To confirm the phenotype of transgenic mice and to explore the AD-like pathologic changes induced by deletion of the gene encoding Nrf2, we performed the immunohistochemical staining of A*β* and p-tauS404. As shown in Figure 2(a), more A*β* plaque deposition was detected in the hippocampus of AT/Nrf2-KO mice than in that of AT mice. The average optical density of anti-A*β* 6E10 positive immunostaining was higher in AT/Nrf2-KO mice than in AT mice (Figure 2(b)). Furthermore, the area of phosphorylated tau-positive cells in CA1 and CA3 of the hippocampus was greater in AT/Nrf2-KO mice than in AT mice (Figures 2(c) and 2(d)). The results suggest that Nrf2 deficiency aggravates A*β* plaque deposition and the expression of p-tauS404 (phosphorylated tau) in AT mice.

### 3.4. Nrf2 Deficiency Aggravates Gliosis in the Hippocampus of AT Mice

**Figure 3:**
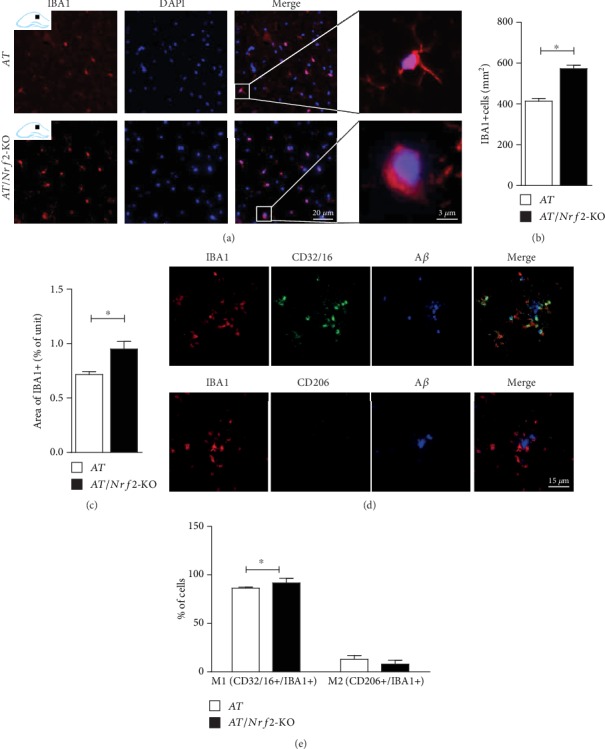
Nrf2 deficiency increased microglia activation in hippocampus of APP1/PS1 mice. (a) Anti-IBA1 immunofluorescence staining in hippocampus CA1 region (scale = 20 *μ*m). (b, c) IBA1 positive cell count (mm^2^) and area of IBA1 positive cells in hippocampus showed significantly between AT and AT/Nrf2-KO genotype mice (*n* = 5/genotype, ^∗^*P* < 0.05). (d) Anti-IBA1, anti-CD32/16, anti-A*β*, anti-IBA1, anti-CD206, and anti-A*β* immunofluorescence triple staining in the hippocampus (scale = 15 *μ*m); the IBA1^+^/CD32/16^+^ cells wrapped the A*β* plaque. (e) The ratio of IBA1^+^/CD32/16^+^ cells surrounding A*β* plaque was significantly increased and in AT/Nrf2-KO mice than that in AT mice (*n* = 5/genotype, ^∗^*P* < 0.05). Data is represented as means ± SD and was analyzed by *t*-test (b, c, e).

We next explored whether the neurological damage associated with the presence or absence of Nrf2 in AT mice was related to the neuroinflammation, comprising activated microglia and astroglia. We used immunofluorescence staining to examine activated microglia through anti-IBA1 and astrogliosis through anti-GFAP. IBA1 expression was increased in the CA1 area of the hippocampus after Nrf2 silencing in AT and AT/Nrf2-KO mice (Figure 3(a)). The microglia underwent essential morphological changes from a branched phenotype to amoeboid cells in AT mice in the presence and absence of Nrf2. We did not observe any difference in microglial process number between these two groups, with most microglial spines seemingly showing no marked differences in morphology after amplification (Figure 3(a), right panel).

The number of IBA1-positive cells and the area of IBA1-positive cells in the hippocampus were greater in AT/Nrf2-KO mice than in AT mice (Figures 3(b) and 3(c)). Next, we examined the M1 proinflammatory phenotype (IBA1^+^ and CD32/16^+^) and M2 anti-inflammatory phenotype (IBA1^+^ and CD206^+^) to evaluate the function of the microglia surrounding the A*β* plaque. As shown in Figure 3(d), M1 phenotype microglia accounted for most of the microglia surrounding the plaque. The ratio of M1-type to M2-type microglia surrounding the plaque was higher in AT/Nrf2-KO mice than in AT mice (Figure 3(e)).

**Figure 4:**
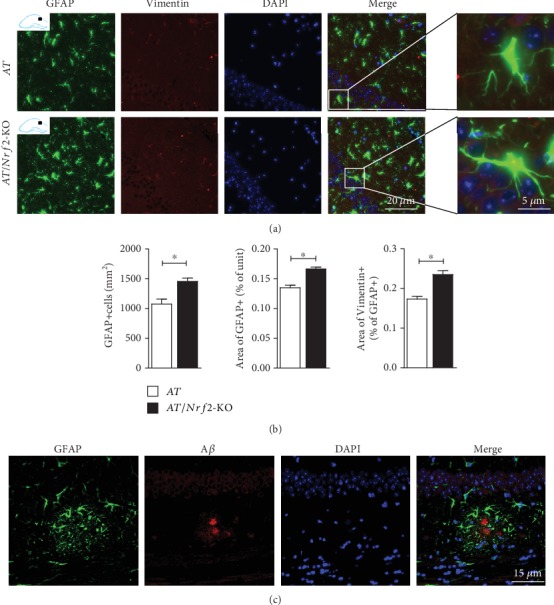
Increased astrocytic activation in hippocampus of Nrf2 deficiency APP1/PS1 mice. (a) Anti-GFAP and anti-Vimentin immunofluorescence double staining in hippocampus CA1 region (scale = 20 *μ*m). (b) GFAP positive cell count (mm^2^), area of GFAP positive cells on per unit, and area of Vimentin positive cells vs. area of GFAP positive cells were significantly different between AT and AT/Nrf2-KO genotype mice in hippocampus (*n* = 5/genotype, ^∗^*P* < 0.05). (c) Anti-GFAP and anti-A*β* immunofluorescence double staining in the hippocampus (scale = 15 *μ*m). Data is represented as means ± SD and was analyzed by *t*-test (b).

In addition, we used GFAP and vimentin antibodies to label reactive astrogliosis. We found that 90.56 ± 0.47% of GFAP-positive cells colocalized with the vimentin signal. As shown in Figure 4(a), GFAP-positive cells were more numerous in the hippocampal CA1 area in AT/Nrf2-KO mice than in AT mice (Figure 4(a)). The reactive astrocytes had more complex and more bulky cell bodies and processes in AT/Nrf2-KO mice than in AT mice (Figure 4(a), right panel). Both the number and area of GFAP-positive cells were increased in the hippocampus (Figure 4(b)). Meanwhile, we explored the relationship between the morphological changes in astrocytes (GFAP^+^) and the A*β* plaque, observing that GFAP-positive astroglia also wrapped around the A*β* deposits, which suggested that the astroglia affected the pathogenesis of A*β* deposition in APP/PS mice (Figure 4(c)).

### 3.5. Nrf2 Deletion Exacerbates Neuroinflammation in the Hippocampus of AT Mice

**Figure 5:**
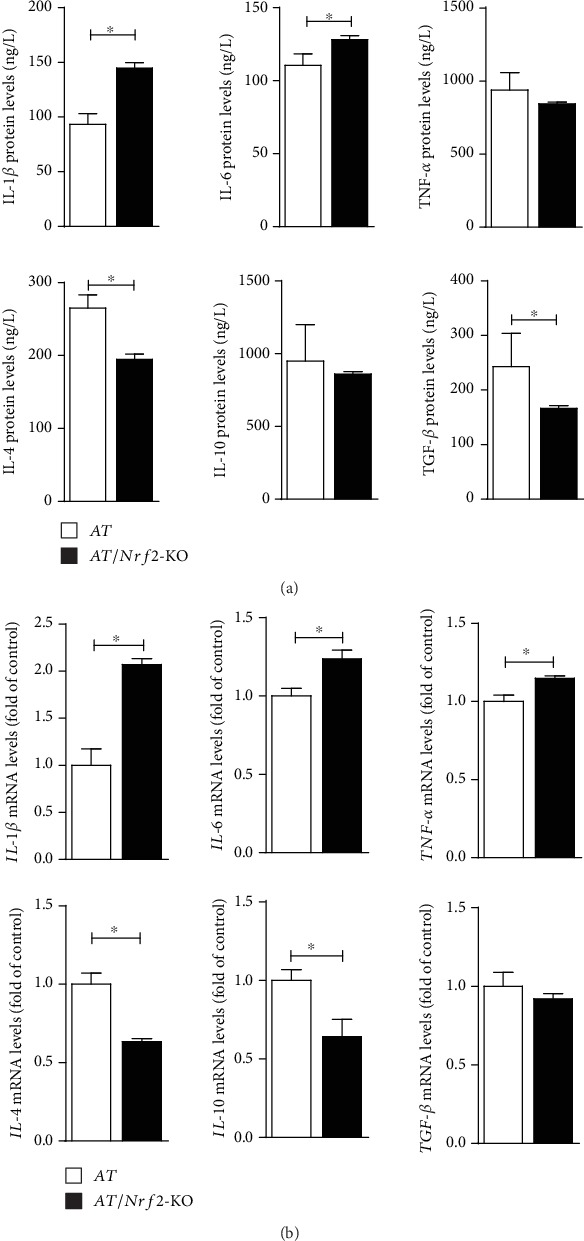
The proinflammatory cytokines protein levels and mRNA levels were increased, and the anti-inflammatory cytokines were reduced in the hippocampus of APP/PS1 mice. (a) Cytokines (IL-1*β*, IL-6, IL-4, and TGF-*β*) in the hippocampus were significantly different between AT and AT/Nrf2-KO genotype mice, analyzed by ELISA (*n* = 5/genotype, ^∗^*P* < 0.05). (b) Levels of mRNA (*IL-1β*, *IL-6*, *TNF-α*, *IL-4*, and *IL-10*) were significantly different between AT and AT/Nrf2-KO genotype mice, determined by qRT-PCR (*n* = 4/genotype, ^∗^*P* < 0.05). Data is represented as means ± SD and was analyzed by *t*-test.

We tested the protein and mRNA levels of proinflammatory cytokines (IL-1*β*, IL-6, and TNF-*α*) and anti-inflammatory cytokines (IL-4, IL-10, and TGF-*β*) by ELISA and qRT-PCR in the hippocampus of AT and AT/Nrf2-KO mice. Overall, both the protein and mRNA levels of IL-1*β*, IL-6, and TNF-*α* were increased in the hippocampus with Nrf2 deletion (Figures 5(a) and 5(b), upper panel). Conversely, the hippocampal protein and mRNA levels of IL-4, IL-10, and TGF-*β* were lower in the AT/Nrf2-KO mice than in the AT/Nrf2-KO mice (Figures 5(a) and 5(b), lower panel), which suggested that Nrf2 deficiency might aggravate the activation of microglia and astrocytes prior to the augmented proinflammatory response in AT mice.

### 3.6. Nrf2 Deletion Aggravates Oxidative Stress Damage in the Hippocampus of AT Mice

**Figure 6:**
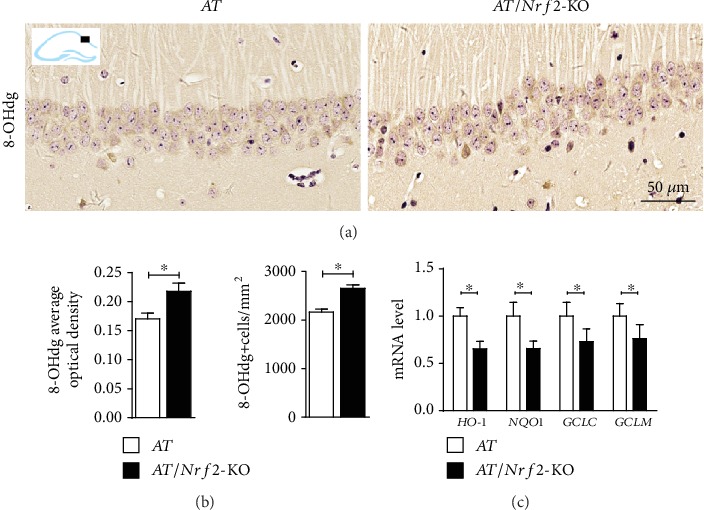
Deletion of Nrf2 in APP/PS1 mice increased oxidative stress damage. (a) Oxidative damage was determined by immunohistochemistry staining with anti-8-OHdg in hippocampus CA1 region (scale = 50 *μ*m). (b) In the AT/Nrf2-KO mice, the average optical density of 8-OHdg in hippocampus and 8-OHdg positive cell count (mm^2^) were significantly higher than AT mice (*n* = 5/genotype, ^∗^*P* < 0.05). (c) The graph showed mRNA levels (*HO-1*, *NQO1*, *GCLC*, and *GCLM*) in the AT and AT/Nrf2-KO genotype mice (*n* = 5/genotype, ^∗^*P* < 0.05). The mRNA levels (*HO-1*, *NQO1*, *GCLC*, and *GCLM*) were significantly reduced in the AT/Nrf2-KO genotype mice. Data is represented as means ± SD and was analyzed by *t*-test (b, c).

Oxidative injury was evaluated by examining the expression of 8-hydroxydeoxyguanosine (8-OHdG) in transgenic mice. We found that 8-OHdG was increased in the hippocampal CA1 in AT/Nrf2-KO mice compared with AT mice (Figure 6(a)).  Figure 6(b) shows that the average optical density of 8-OHdG in the hippocampal CA1 was also higher in Nrf2-deficient AT mice. Moreover, we determined that the mRNA levels of several classical antioxidant enzymes were regulated by the Nrf2-ARE pathway. *HO-1*, *NQO1*, *GCLC*, and *GCLM* mRNA levels were decreased in AT/Nrf2-KO mice compared with AT/Nrf2-KO mice (Figure 6(c)). Our results suggest that the oxidative stress damage induced by APP/PS1 expression is increased in Nrf2-deficient AT mice.

## 4. Discussion

AD is an irreversible chronic neurodegenerative disease characterized by senile plaques and NFTs. Extensive evidence supports various possible etiologies of AD, which include oxidative stress, inflammation, autophagy, tau and amyloid toxicity, and genetic mutations [5, 24, 25]. In addition, considerable data suggest that Nrf2 activity is impaired or insufficient during AD pathogenesis, which is associated with oxidative stress, inflammation, and autophagy [26, 27]. Nrf2 is an important antioxidant stress and anti-inflammatory factor that plays a protective role in the AD brain [27, 28]. A recent transcriptomic study revealed that Nrf2 might regulate other multiple stress responses in Nrf2 knockout AD mice [14]. In the present study, we found increased expression of Nrf2 in the hippocampus of AT mice, where it had an abnormal transcriptional regulation function. Gene deletion of Nrf2 exacerbated the AD-like pathologic changes in AT mice by worsening the neuroinflammation and oxidative stress.

### 4.1. Nrf2 Is an Important Regulator in the Pathogenesis of AD

Nrf2 is widely expressed in the brain. Some evidence indicates that endogenous Nrf2 is readily inducible and tends to be more strongly activated in neurons in AD [18, 29–31]. Here, we found that Nrf2 was upregulated in the hippocampus, which is in line with results showing that Nrf2 is elevated in the hippocampal cells of AD brain tissue [18, 29–31]. We also discovered with immunohistochemical staining that most Nrf2 was highly located in the cytosol of the neurons and weakly expressed in their nuclei, which is consistent with previous results from the Jordan-Sciutto laboratory [11]. Indeed, it is interesting that different research groups have revealed different patterns of Nrf2 expression in AD brain tissue from human and animal models. Koistinaho and colleagues [20] reported an apparent decrease in Nrf2 activity in hippocampal neurons and the frontal cortex of a transgenic AD mouse model through immunohistochemical staining and western blot of nuclear fractions. Although Nrf2 activity varies among animal models and regions, it is clear that endogenous Nrf2 activity is insufficient to prevent oxidative stress and neuronal cell dysfunction in AD disease.

Accumulating genetic and pharmaceutical studies have shown that Nrf2 plays a neuroprotective role in the AD-related pathophysiology [19, 20, 32, 33]. Overexpression of Nrf2 specifically in hippocampal neurons lessens astrocyte activation and alleviates cognitive dysfunction without diminishing A*β* pathology in AT mice [33], suggesting that Nrf2 activation in neurons is sufficient to prevent neuronal dysfunction in AD. Deletion or mutation of Nrf2 worsens A*β* pathology [18, 29], as well as tau pathology and learning and memory impairments [14]. Nrf2 has also been reported to protect neural progenitor cells [34] and PC12 cells [35] against A*β* toxicity in AD. High expression of Nrf2 decreases the A*β*-induced oxidative stress and neurotoxicity, which prevents and delays AD-like pathology [20]. Hydrogen sulfide inhibits A*β* production and decreases senile plaque aggregation by activating Nrf2 [36]. In addition, methylene blue ameliorates the behavioral anomaly and reduces the phosphorylated tau pathology by upregulating the Nrf2-ARE pathway [7]. In contrast, the expression of phosphorylated tau and sarkosyl-insoluble tau are increased in Nrf2-KO mouse [37]. Our data show that A*β* and p-tauS404 were increased in the hippocampus of Nrf2 knockout AT mice compared with AT mice and that Nrf2 deficiency aggravated the spatial learning and memory ability impairment in AT mice, which is in line with previous results [14, 21, 33]. Our study suggests that Nrf2 might be involved in A*β* deposition and phosphorylation of tau and play neuroprotective roles in AD animal models.

### 4.2. Inflammatory Response in the Process of AD: The Role of Nrf2

Although there is overwhelming evidence for a pathogenic role for A*β* and tau hyperphosphorylation in AD, neuroinflammation is widely regarded as the key mechanism that actively contributes to the pathology by modulating the responses of microglia and astrocytes [38–43]. This belief is also supported by evidence that anti-inflammatory treatments delay AD onset and alleviate or slow the cognitive decline [44]. Data from Yamamoto et al. [9] proved that Nrf2 attenuates inflammation by opposing transcriptional upregulation of the proinflammatory cytokine genes IL-6 and IL-1*β* rather than by inhibiting oxidative stress. Previous work shows that Nrf2-knockout mice crossed with AT or mutant HsAPPV717I/HsMAPTP301L mice exhibit exacerbated astrocyte and microglial activation [14, 18, 29]. In addition, the Nrf2 activator methysticin significantly reduces microglial infiltration, astrogliosis, and the secretion of the proinflammatory cytokines TNF-*α* and IL-17A in APP/Psen1 mice [45]. In the present study, deletion of Nrf2 exacerbated the inflammatory response in AT mice, indicated by upregulated proinflammatory factors and hippocampal astrogliosis and activation of microglia, especially those surrounding the A*β* plaque, which is consistent with previous studies showing that activated microglia appear around the NFTs (neurofibrillary tangles) and A*β* plaques in AD animal models or human patients [21, 46–48]. Indeed, both hyperphosphorylated tau protein [49] and fibrillar A*β* [50] lead to inflammatory processes and the release of IL-1*β* in vivo and in vitro. Although appropriate inflammation could stimulate microglia to promote the phagocytic removal of A*β* deposits, microglia can exert both benign and damaging activities in AD. With the accumulation of A*β*, microglial cells may become progressively impaired in their ability to phagocytize A*β* [51, 52]. We found that overwhelmingly predominant M1 proinflammatory microglia had infiltrated and surrounded A*β* plaques instead of M2 anti-inflammatory microglia in the hippocampus of AT mice without Nrf2, suggesting that the excessive production of inflammatory cytokines or mediators by activated M1 microglia cells might be the main reason for the aggravation of the neurological damage in AT/Nrf2-KO mice. Astrocytes participate in inflammatory processes during AD, as we showed in the present study, and the absence of Nrf2 induced more aggressive activation of astroglia and inflammation in AT mice, which might be through activation of the NF-*κ*B pathway [53, 54].

### 4.3. Nrf2 Plays Neuroprotective Role in AD through Regulating Antioxidant Response

Nrf2 is a key regulator of the antioxidant response, which regulates cytoprotective and detoxificant genes and plays a neuroprotective role in AD [55, 56]. An Nrf2 target gene, Nqo1, is increased in the hippocampus [57, 58] and frontal cortex (although less strongly than in the hippocampus) [31], whereas HO-1 is increased in the temporal cortex and hippocampus of AD human brains [29, 30]. Hippocampal expression of mutated tau elevates HO-1 and GCLC transcripts in WT but not Nrf2 knockout mice [30], suggesting that the antioxidant response is Nrf2 dependent. According to the present data, the level of Nrf2 is increased in AT mice, but the cytosolic accumulation and insufficient antioxidant response increase the oxidative stress damage in the hippocampus of AT mice. Nrf2 ablation aggravated oxidative damage in the hippocampus of AT mice, reflected by the upregulated immunoreactivity of 8-OHdG, suggesting a protective role for the Nrf2-mediated antioxidant response in AT mice. 8-OHdG is generally regarded as a biomarker of mutagenesis caused by oxidative stress because it is a product of aerobic metabolism when DNA is attacked by oxidative stress-related hydroxyl radicals. Thus, 8-OHdG represents the extent of oxidative DNA damage. Collectively, these data show that Nrf2 deficiency increases oxidative stress in AT mice by weakening the expression of its downstream antioxidant genes.

### 4.4. Limitations of the Study

In the present study, Nrf2 was only shown to participate in the pathogenesis of AD by inhibiting inflammation and oxidative stress; other hypotheses were not investigated. In addition, we only used Nrf2-KO mice, although it would be better to use Nrf2 activators for further study. Finally, the model involved the global knockout of Nrf2, that is, both in neurons and different glia, and it is difficult to determine its cell-specific function in the pathogenesis of AD.

### 4.5. Prospect

Oxidative damage and neuroinflammation are the central mechanisms in the pathogenesis of AD [37, 58]. One way to render neuronal cells more resistant to oxidative stress and inflammation is to upregulate the endogenous protection system, including antioxidant factors and anti-inflammatory cytokines. The present study, consistent with previous work, proved that Nrf2 might be a good way to ameliorate AD-related cognitive impairment and AD-like pathology changes, which is worth exploring in the clinical setting.

## 5. Conclusion

In summary, the present data show that Nrf2 ablation promotes AD-like pathology in AT mice. Deletion of Nrf2 aggravated the damage caused by neuroinflammation and oxidative stress, suggesting a key protective role for Nrf2 in the pathogenesis of AD. In addition, Nrf2 might serve as a good target for the treatment of AD in the future.

## Figures and Tables

**Table 1:** Antibodies used for IHC and IF.

Primary antibodies	Rabbit anti-Nrf2 polyclonal antibody (1 : 400 dilution, ab62352; Abcam)
	Mouse anti-A*β* clone 6E10 antibody (1 : 400 dilution, 803001; Biolegend)
	Rabbit anti-p-tauS404 monoclonal antibody (1 : 400 dilution, ab92676; Abcam)
	Goat anti-8-OHdG polyclonal antibody (1 : 200 dilution, ab10802; Abcam)
	Goat anti-CD32/16 (1 : 200 dilution, AF1460; R&D Systems)
	Goat anti-CD206 polyclonal antibody (1 : 200 dilution, PA5-46994; Invitrogen)
	Rabbit anti-IBA1 polyclonal antibody (1 : 200 dilution, 10904-1-AP; ProteinTech)
	Rabbit anti-GFAP monoclonal antibody (1 : 200 dilution, 60190-1-lg; ProteinTech)
	Goat anti-Vimentin polyclonal antibody (1 : 200 dilution, ab11256; Abcam)

Secondary antibodies	Alexa Fluor® 488 donkey anti-rabbit IgG (1 : 400 dilution, A21206; Invitrogen)
	Alexa Fluor® 594 donkey anti-goat IgG (1 : 400 dilution, A32758; Invitrogen)
	Alexa Fluor® 594 donkey anti-mouse IgG (1 : 400 dilution, A21203; Invitrogen)
	Alexa Fluor® 594 donkey anti-rabbit IgG (1 : 400 dilution, A21207; Invitrogen)
	Alexa Fluor® 488 donkey anti-goat IgG (1 : 400 dilution, A32814; Invitrogen)
	Alexa Fluor® 350 donkey anti-mouse IgG (1 : 400 dilution, A10035; Invitrogen)

**Table 2:** Primer sequences used for qRT-PCR.

Gene	Forward	Reverse
Nrf2	TCCCATTTGTAGATGACCATGAG	CCATGTCCTGCTCTATGCTG
IL-1*β*	ACGGACCCCAAAAGATGAAG	TTCTCCACAGCCACAATGAG
IL-4	CGAATGTACCAGGAGCCATATC	TCTCTGTGGTGTTCTTCGTTG
IL-6	CAAAGCCAGAGTCCTTCAGAG	GTCCTTAGCCACTCCTTCTG
IL-10	AGCCGGGAAGACAATAACTG	GGAGTCGGTTAGCAGTATGTTG
TNF-*α*	CTTCTGTCTACTGAACTTCGGG	CAGGCTTGTCACTCGAATTTTG
TGF-*β*	CCTGAGTGGCTGTCTTTTGA	CGTGGAGTTTGTTATCTTTGCTG
HO-1	ACAGAGGAACACAAAGACCAG	GTGTCTGGGATGAGCTAGTG
NQO1	TGAAGAAGAGAGGATGGGAGG	GATGACTCGGAAGGATACTGAAAG
GCLC	ACCATCACTTCATTCCCCAG	TTCTTGTTAGAGTACCGAAGCG
GCLM	AATCAGCCCCGATTTAGTCAG	CGATCCTACAATGAACAGTTTTGC
GAPDH	CTTTGTCAAGCTCATTTCCTGG	TCTTGCTCAGTGTCCTTGC

## Data Availability

The data used to support the findings of this study are available from the corresponding author upon request.

## Supplementary Materials

Supplementary Materials Figure S1: Enhanced level of Nrf2 in the hippocampus in the APP/PS1 mice. (A) Nrf2 mice genotype (upper, *n* = 3/genotype) and APP/PS1 mice genotype (lower, *n* = 3/genotype). (B) Immunohistochemical staining with anti-Nrf2 in hippocampus CA1, CA3, and DG in the WT (APP/PS1^−/−^, Nrf2^+/−^) and AT (APP/PS1^+/−^, Nrf2^+/−^) genotype mice (scale = 50 *μ*m). (C) Average optical density of Nrf2 immunoreactivity in hippocampus was significantly different between WT (APP/PS1^−/−^, Nrf2^+/−^) and AT (APP/PS1^+/−^, Nrf2^+/−^) genotype mice (*n* = 3/genotype, ^∗^*P* < 0.05). (D) qRT-PCR determination of hippocampus Nrf2 mRNA levels showed significantly between WT (APP/PS1^−/−^, Nrf2^+/−^) and AT (APP/PS1^+/−^, Nrf2^+/−^) genotype mice (*n* = 5/genotype, ^∗^*P* < 0.05). Data is represented as means ± SD and were analyzed by *t*-test (C, D). Table S1: We generated four genotypes (WT, Nrf2-KO, AT, and AT/Nrf2-KO). The number represented the number of mice with different genotypes in the corresponding experiments. MWM: Morris water maze test; IHC/IF: immunohistochemistry/immunofluorescence; qRT-PCR: quantitative real-time PCR; ELISA: enzyme-linked immunosorbent assay; M: male; F: female; WT: wild-type mice (APP/PS1^−/−^, Nrf2^+/−^); Nrf2-KO: Nrf2-knockout mice (APP/PS1^−/−^, Nrf2^−/−^); AT: APP/PS1 transgenic mice (APP/PS1^+/−^, Nrf2^+/−^); AT/Nrf2-KO: APP/PS1/Nrf2-knockout mice (APP/PS1^+/−^, Nrf2^−/−^).

## References

[1] Huang Y., Mucke L. (2012). Alzheimer mechanisms and therapeutic strategies. *Cell*.

[2] Šimić G., Babić Leko M., Wray S. (2016). Tau protein hyperphosphorylation and aggregation in Alzheimer’s disease and other tauopathies, and possible neuroprotective strategies. *Biomolecules*.

[3] Gouras G. K., Almeida C. G., Takahashi R. H. (2005). Intraneuronal A*β* accumulation and origin of plaques in Alzheimer's disease. *Neurobiology of Aging*.

[4] Galimberti D., Scarpini E. (2011). Inflammation and oxidative damage in Alzheimer s disease friend or foe. *Frontiers in Bioscience*.

[5] Prasad K. N. (2017). Oxidative stress and pro-inflammatory cytokines may act as one of the signals for regulating microRNAs expression in Alzheimer’s disease. *Mechanisms of Ageing and Development*.

[6] Haass C., Selkoe D. J. (2007). Soluble protein oligomers in neurodegeneration: lessons from the Alzheimer's amyloid *β*-peptide. *Nature Reviews Molecular Cell Biology*.

[7] Stack C., Jainuddin S., Elipenahli C. (2014). Methylene blue upregulates Nrf 2/ARE genes and prevents tau-related neurotoxicity. *Human Molecular Genetics*.

[8] Li L., Dong H., Song E., Xu X., Liu L., Song Y. (2014). Nrf2/ARE pathway activation, HO-1 and NQO1 induction by polychlorinated biphenyl quinone is associated with reactive oxygen species and PI3K/AKT signaling. *Chemico-Biological Interactions*.

[9] Kobayashi E. H., Suzuki T., Funayama R. (2016). Nrf2 suppresses macrophage inflammatory response by blocking proinflammatory cytokine transcription. *Nature Communications*.

[10] Zhang H., Davies K. J. A., Forman H. J. (2015). Oxidative stress response and Nrf2 signaling in aging. *Free Radical Biology and Medicine*.

[11] Ramsey C. P., Glass C. A., Montgomery M. B. (2007). Expression of Nrf 2 in neurodegenerative diseases. *Journal of Neuropathology & Experimental Neurology*.

[12] Markesbery W. R., Carney J. M. (1999). Oxidative alterations in Alzheimer's disease. *Brain Pathology*.

[13] Branca C., Ferreira E., Nguyen T. V., Doyle K., Caccamo A., Oddo S. (2017). Genetic reduction of Nrf 2 exacerbates cognitive deficits in a mouse model of Alzheimer's disease. *Human Molecular Genetics*.

[14] Rojo A. I., Pajares M., Rada P. (2017). NRF2 deficiency replicates transcriptomic changes in Alzheimer's patients and worsens APP and TAU pathology. *Redox Biology*.

[15] Komatsu M., Kurokawa H., Waguri S. (2010). The selective autophagy substrate p 62 activates the stress responsive transcription factor Nrf 2 through inactivation of Keap 1. *Nature Cell Biology*.

[16] Lau A., Wang X. J., Zhao F. (2010). A noncanonical mechanism of Nrf 2 activation by autophagy deficiency: direct interaction between Keap 1 and p 62. *Molecular and Cellular Biology*.

[17] Riley B. E., Kaiser S. E., Kopito R. R. (2014). Autophagy inhibition engages Nrf 2-p 62 Ub-associated signaling. *Autophagy*.

[18] Joshi G., Gan K. A., Johnson D. A., Johnson J. A. (2015). Increased Alzheimer's disease–like pathology in the APP/ PS1ΔE9 mouse model lacking Nrf2 through modulation of autophagy. *Neurobiology of Aging*.

[19] Bahn G., Jo D. G. (2019). Therapeutic approaches to Alzheimer's disease through modulation of NRF2. *Neuromolecular Medicine*.

[20] Kanninen K., Malm T. M., Jyrkkänen H. K. (2008). Nuclear factor erythroid 2-related factor 2 protects against beta amyloid. *Molecular and Cellular Neuroscience*.

[21] Lok K., Zhao H., Shen H. (2013). Characterization of the APP/PS1 mouse model of Alzheimer's disease in senescence accelerated background. *Neuroscience Letters*.

[22] Caccamo A., Branca C., Talboom J. S. (2015). Reducing ribosomal protein S6 kinase 1 expression improves spatial memory and synaptic plasticity in a mouse model of Alzheimer's disease. *Journal of Neuroscience*.

[23] Dong W., Yang B., Wang L. (2018). Curcumin plays neuroprotective roles against traumatic brain injury partly via Nrf2 signaling. *Toxicology and Applied Pharmacology*.

[24] Schellenberg G. D., Montine T. J. (2012). The genetics and neuropathology of Alzheimer's disease. *Acta Neuropathologica*.

[25] Guo J., Cheng J., North B. J., Wei W. (2017). Functional analyses of major cancer-related signaling pathways in Alzheimer's disease etiology. *Biochimica et Biophysica Acta (BBA) - Reviews on Cancer*.

[26] Zhang M., Teng C.‑. H., Wu F.‑. F. (2019). Edaravone attenuates traumatic brain injury through anti-inflammatory and anti-oxidative modulation. *Experimental and Therapeutic Medicine*.

[27] Pajares M., Jiménez-Moreno N., García-Yagüe Á. J. (2016). Transcription factor NFE2L2/NRF2 is a regulator of macroautophagy genes. *Autophagy*.

[28] Sivandzade F., Bhalerao A., Cucullo L. (2019). Cerebrovascular and neurological disorders: protective role of NRF2. *International Journal of Molecular Sciences*.

[29] Lastres-Becker I., Innamorato N. G., Jaworski T. (2014). Fractalkine activates NRF2/NFE2L2 and heme oxygenase 1 to restrain tauopathy-induced microgliosis. *Brain*.

[30] SantaCruz K. S., Yazlovitskaya E., Collins J., Johnson J., DeCarli C. (2004). Regional NAD(P)H:quinone oxidoreductase activity in Alzheimer’s disease. *Neurobiology of Aging*.

[31] Liddell J. (2017). Are astrocytes the predominant cell type for activation of Nrf 2 in aging and neurodegeneration?. *Antioxidants*.

[32] Kanninen K., Heikkinen R., Malm T. (2009). Intrahippocampal injection of a lentiviral vector expressing Nrf 2 improves spatial learning in a mouse model of Alzheimer's disease. *Proceedings of the National Academy of Sciences*.

[33] Kärkkäinen V., Pomeshchik Y., Savchenko E. (2014). Nrf2 regulates neurogenesis and protects neural progenitor cells against A*β* toxicity. *Stem Cells*.

[34] Wruck C. J., Götz M. E., Herdegen T., Varoga D., Brandenburg L. O., Pufe T. (2008). Kavalactones protect neural cells against amyloid *β* peptide-induced neurotoxicity via extracellular signal-regulated kinase 1/2-dependent nuclear factor erythroid 2-related factor 2 activation. *Molecular Pharmacology*.

[35] Liu Y., Deng Y., Liu H., Yin C., Li X., Gong Q. (2016). Hydrogen sulfide ameliorates learning memory impairment in APP/PS1 transgenic mice: A novel mechanism mediated by the activation of Nrf2. *Pharmacology, Biochemistry, and Behavior*.

[36] Jo C., Gundemir S., Pritchard S., Jin Y. N., Rahman I., Johnson G. V. W. (2014). Nrf2 reduces levels of phosphorylated tau protein by inducing autophagy adaptor protein NDP52. *Nature Communications*.

[37] Bolos M., Perea J. R., Avila J. (2017). Alzheimer's disease as an inflammatory disease. *Biomolecular concepts*.

[38] Zhang D., Lu Z., Man J. (2019). Wnt-3a alleviates neuroinflammation after ischemic stroke by modulating the responses of microglia/macrophages and astrocytes. *International Immunopharmacology*.

[39] Facci L., Barbierato M., Skaper S. D. (2018). Astrocyte/microglia cocultures as a model to study neuroinflammation. *Methods Molecular Biology*.

[40] McGeer P. L., McGeer E. G. (2013). The amyloid cascade-inflammatory hypothesis of Alzheimer disease: implications for therapy. *Acta Neuropathologica*.

[41] De Strooper B., Karran E. (2016). The Cellular Phase of Alzheimer’s Disease. *Cell*.

[42] Sala F. C., De Strooper B. (2016). Alzheimer's disease mechanisms and emerging roads to novel therapeutics. *Annual Review of Neuroscience*.

[43] Broussard G. J., Mytar J., Li R. C., Klapstein G. J. (2012). The role of inflammatory processes in Alzheimer's disease. *Inflammopharmacology*.

[44] Singhal G., Jaehne E. J., Corrigan F., Toben C., Baune B. T. (2014). Inflammasomes in neuroinflammation and changes in brain function: a focused review. *Frontiers in Neuroscience*.

[45] Rojo A. I., Innamorato N. G., Martín-Moreno A. M., de Ceballos M. L., Yamamoto M., Cuadrado A. (2010). Nrf 2 regulates microglial dynamics and neuroinflammation in experimental Parkinson's disease. *Glia*.

[46] Griffin W. S., Stanley L. C., Ling C. (1989). Brain interleukin 1 and S-100 immunoreactivity are elevated in Down syndrome and Alzheimer disease. *Proceedings of the National Academy of Sciences*.

[47] Simard A. R., Rivest S. (2006). Neuroprotective properties of the innate immune system and bone marrow stem cells in Alzheimer's disease. *Molecular Psychiatry*.

[48] Licht-Murava A., Paz R., Vaks L. (2016). A unique type of GSK-3 inhibitor brings new opportunities to the clinic. *Science Signaling*.

[49] Halle A., Hornung V., Petzold G. C. (2008). The NALP3 inflammasome is involved in the innate immune response to amyloid-*β*. *Nature Immunology*.

[50] Sarlus H., Heneka M. T. (2017). Microglia in Alzheimer's disease. *Journal of Clinical Investigation*.

[51] Shi Y., Holtzman D. M. (2018). Interplay between innate immunity and Alzheimer disease: APOE and TREM2 in the spotlight. *Nature Reviews Immunology*.

[52] Pan H., Wang H., Zhu L., Mao L., Qiao L., Su X. (2011). Depletion of Nrf 2 enhances inflammation induced by oxyhemoglobin in cultured mice astrocytes. *Neurochemical Research*.

[53] Fragoulis A., Laufs J., Müller S. (2012). Sulforaphane has opposing effects on TNF-alpha stimulated and unstimulated synoviocytes. *Arthritis Research & Therapy*.

[54] Buendia I., Michalska P., Navarro E., Gameiro I., Egea J., León R. (2016). Nrf2–ARE pathway: An emerging target against oxidative stress and neuroinflammation in neurodegenerative diseases. *Pharmacology & Therapeutics*.

[55] Ma Q. (2013). Role of Nrf 2 in oxidative stress and toxicity. *Annual review of pharmacology and toxicology*.

[56] Raina A. K., Templeton D. J., Deak J. C., Perry G., Smith M. A. (2013). Quinone reductase (NQO1), a sensitive redox indicator, is increased in Alzheimer's disease. *Redox Report*.

[57] Wang Y., Santa–Cruz K., DeCarli C., Johnson J. A. (2000). NAD(P)H:quinone oxidoreductase activity is increased in hippocampal pyramidal neurons of patients with alzheimer’s disease. *Neurobiology of Aging*.

[58] Butterfield D. A., Reed T., Newman S. F., Sultana R. (2007). Roles of amyloid *β*-peptide-associated oxidative stress and brain protein modifications in the pathogenesis of Alzheimer's disease and mild cognitive impairment. *Free Radical Biology and Medicine*.

