# Supplementary material for: Nrf2 Ablation Promotes Alzheimer's Disease-Like Pathology in APP/PS1 Transgenic Mice: The Role of Neuroinflammation and Oxidative Stress
Source: Oxid Med Cell Longev. 2020 May 11;2020:3050971. doi: 10.1155/2020/3050971 (PMC7238335; doi:10.1155/2020/3050971)
Supplement: Supplementary Materials — Figure S1: Enhanced level of Nrf2 in the hippocampus in the APP/PS1 mice. (A) Nrf2 mice genotype (upper, n = 3/genotype) and APP/PS1 mice genotype (lower, n = 3/genotype). (B) Immunohistochemical staining with anti-Nrf2 in hippocampus CA1, CA3, and DG in the WT (APP/PS1−/−, Nrf2+/−) and AT (APP/PS1+/−, Nrf2+/−) genotype mice (scale = 50 μm). (C) Average optical density of Nrf2 immunoreactivity in hippocampus was significantly different between WT (APP/PS1−/−, Nrf2+/−) and AT (APP/PS1+/−, Nrf2+/−) genotype mice (n = 3/genotype, ∗P < 0.05). (D) qRT-PCR determination of hippocampus Nrf2 mRNA levels showed significantly between WT (APP/PS1−/−, Nrf2+/−) and AT (APP/PS1+/−, Nrf2+/−) genotype mice (n = 5/genotype, ∗P < 0.05). Data is represented as means ± SD and were analyzed by t-test (C, D). Table S1: We generated four genotypes (WT, Nrf2-KO, AT, and AT/Nrf2-KO). The number represented the number of mice with different genotypes in the corresponding experiments. MWM: Morris water maze test; IHC/IF: immunohistochemistry/immunofluorescence; qRT-PCR: quantitative real-time PCR; ELISA: enzyme-linked immunosorbent assay; M: male; F: female; WT: wild-type mice (APP/PS1−/−, Nrf2+/−); Nrf2-KO: Nrf2-knockout mice (APP/PS1−/−, Nrf2−/−); AT: APP/PS1 transgenic mice (APP/PS1+/−, Nrf2+/−); AT/Nrf2-KO: APP/PS1/Nrf2-knockout mice (APP/PS1+/−, Nrf2−/−). [file 3050971.f1.pdf]

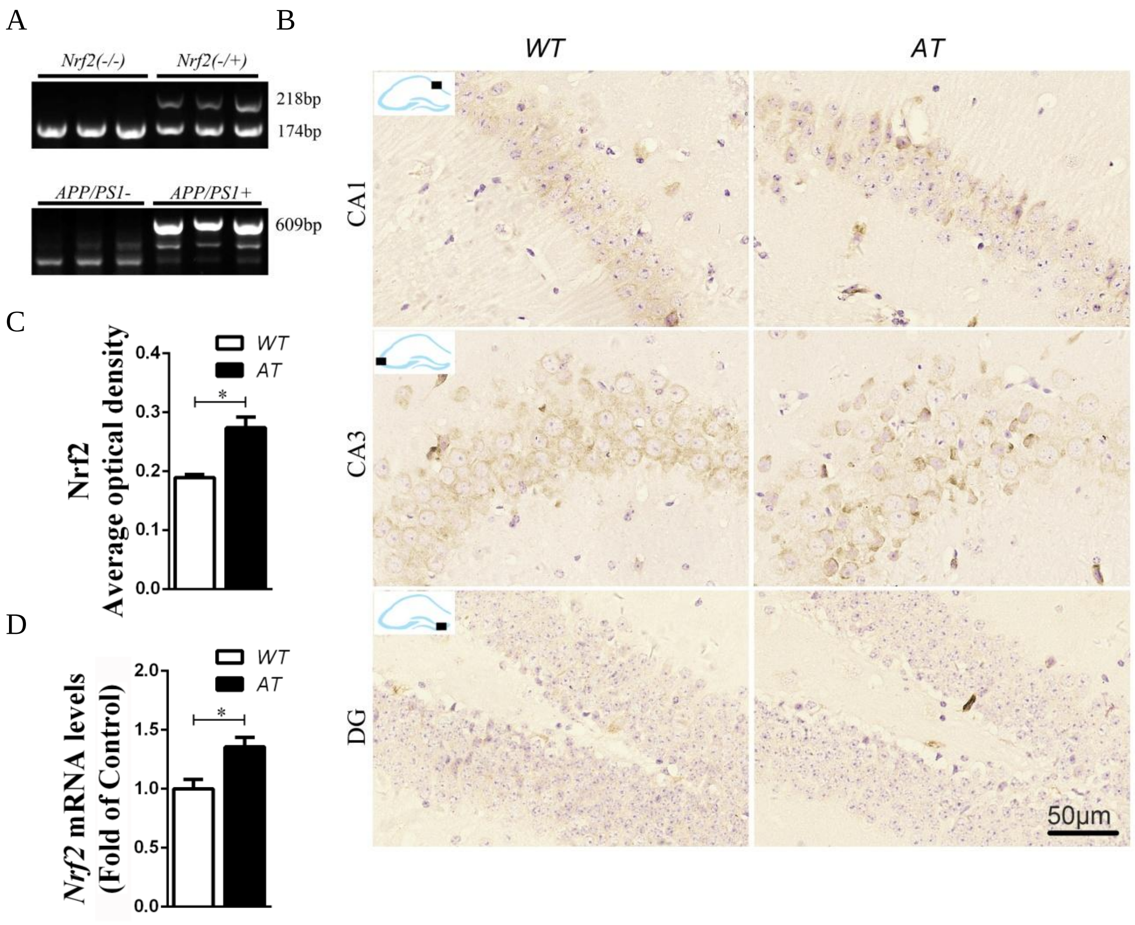

Figure S1: Enhanced level of Nrf2 in the hippocampus in the APP/PS1 mice. (A) Nrf2 mice genotype (upper, n=3/genotype) and APP/PS1 mice genotype (lower, n=3/genotype). (B) Immumohistochemical staining with anti-Nrf2 in hippocampus CA1, CA3 and DG in the WT (APP/PS1$^{-/-}$, Nrf2$^{+/-}$) and AT (APP/PS1$^{+/-}$, Nrf2$^{+/-}$) genotype mice (scale=50μm). (C) Average optical density of Nrf2 immunoreactivity in hippocampus was significantly different between WT (APP/PS1$^{-/-}$, Nrf2$^{+/-}$) and AT (APP/PS1$^{+/-}$, Nrf2$^{+/-}$) genotype mice (n=3/genotype, *$P < 0.05$). (D) qRT-PCR determination of hippocampus Nrf2 mRNA levels showed significantly between WT (APP/PS1$^{-/-}$, Nrf2$^{+/-}$) and AT (APP/PS1$^{+/-}$, Nrf2$^{+/-}$) genotype mice (n=5/genotype, *$P < 0.05$). Data is represented as means±SD and were analysed by t-test (C, D).

Table.S1 The information of mice and their use for various protocols

	MWM		IHC/IF		qRT-PCR		ELISA	
	M	F	M	F	M	F	M	F
WT	4	2	4	2	4	2	0	0
Nrf2-KO	6	2	4	2	4	2	0	0
AT	6	6	3	3	3	3	3	2
AT/*Nrf2*-KO	6	7	3	3	3	3	2	3

Table S1: We generated four genotypes (WT, Nrf2-KO, AT and AT/Nrf2-KO). The number represented the number of mice with different genotypes in the corresponding expriments. MWM: Morris water maze test; IHC/IF: Immunohistochemistry/ immunofluorescence; qRT-PCR: quantitative real-time PCR; ELISA: enzyme-linked immunosorbent assay; M: male; F: female; WT: wild-type mice (APP/PS1$^{-/-}$, Nrf2$^{+/-}$); Nrf2-KO: Nrf2-knockout mice (APP/PS1$^{-/-}$, Nrf2$^{-/-}$); AT: APP/PS1 transgenic mice (APP/PS1$^{+/-}$, Nrf2$^{+/-}$); AT/Nrf2-KO: APP/PS1/Nrf2-knockout mice (APP/PS1$^{+/-}$, Nrf2$^{-/-}$).
